# Neoadjuvant immunotherapy in breast cancer: a paradigm shift?

**DOI:** 10.3332/ecancer.2020.1147

**Published:** 2020-12-03

**Authors:** Elena Fountzila, Michail Ignatiadis

**Affiliations:** 1 European University Cyprus, German Oncology Center, Agios Athanasios, 22006, Cyprus; 2 Second Department of Medical Oncology, Euromedica General Clinic, Thessaloniki, 54645, Greece; 3 Department of Medical Oncology & Academic Trials Promoting Team, Institut Jules Bordet, Université Libre de Bruxelles, Bruxelles, 1000, Belgium

**Keywords:** biomarker, clinical trial, immunotherapy, neoadjuvant, pathologic complete response, triple-negative breast cancer

## Abstract

Despite advances in clinical management, a proportion of patients with early-stage triple-negative breast cancer (TNBC) recur after local treatment. The concept of neoadjuvant systemic therapy has been widely adopted to improve clinical outcomes of patients with TNBC and other breast tumour types. Recently, promising data were reported from the first prospective phase III, randomised trial assessing neoadjuvant chemotherapy combined with the programmed cell death protein 1 (PD-1) inhibitor pembrolizumab versus placebo in patients with early-stage TNBC. The addition of pembrolizumab resulted in a significant increase in pathologic complete response (pCR) rates. Similarly, in the IMpassion031 trial, the use of atezolizumab in combination with neoadjuvant chemotherapy in patients with early-stage TNBC led to improved pCR rates compared to placebo, regardless of programmed death ligand 1 (PD-L1) expression. Ongoing trials are testing other PD-1/PD-L1 inhibitors in combination with neoadjuvant chemotherapy in TNBC and other tumour subtypes. However, not all patients benefit from the addition of immunotherapy, while a proportion of patients experiences serious adverse events. It is critical to identify predictive biomarkers of response, to accurately select patients who will benefit from immunotherapy, thus sparing the rest from ineffective treatments with unnecessary toxicity and treatment costs. In this review, we summarise the literature on reported and ongoing neoadjuvant clinical trials evaluating immunotherapy in breast cancer.

## Introduction

Despite advances in clinical management, a proportion of patients with early-stage breast cancer recur after local treatment. Recurrence rates are higher in HER2-positive or triple-negative disease, and prognosis of patients with advanced cancer remains poor [1, 2]. Thus, there is an unmet need to improve therapeutic management of early-stage disease to decrease the likelihood of recurrence. Adjuvant chemotherapy with standard regimens, including anthracyclines, taxanes and cyclophosphamide was the preferred therapeutic option for patients with operable breast cancer. Recently, the concept of pre-operative (neoadjuvant) systemic therapy has been increasingly used to improve the clinical management of patients with breast cancer. First, the use of neoadjuvant therapy may lead to a significant decrease in the tumour and/or lymph node load and facilitate the subsequent surgical procedures [3, 4]. In addition, this approach provides informative data regarding prognosis and response to systemic interventions. Pathologic complete response (pCR) in the breast and axillary nodes noted at the time of surgery following neoadjuvant treatment has been associated with improved clinical outcomes, particularly in triple-negative and HER2-positive diseases [5–9]. Thus, it has been accepted by the U.S. Food and Drug Administration as a surrogate endpoint to accelerate drug approval [10]. Finally, based on recently published data, the addition of chemotherapeutic agents as adjuvant treatment in patients with breast cancer who did not achieve pCR after neoadjuvant therapy should be considered [11, 12]. Various therapeutic agents are currently being evaluated in the neoadjuvant setting [13–16].

In the evolving era of immunotherapy, several clinical trials are focusing on the evaluation of immunotherapeutic agents at the neoadjuvant setting in patients with triple-negative breast cancer (TNBC) [17–21]. These attempts are supported by promising data on the use of immunotherapy in patients with advanced triple-negative disease. In a recently published phase III trial, the administration of the programmed death-ligand 1 (PD-L1) inhibitor atezolizumab in combination with chemotherapy led to improved progression-free survival (PFS) compared to chemotherapy alone as first-line treatment in patients with metastatic TNBC [22]. The rationale of using neoadjuvant immunotherapy lies, among others, on the potential increase of systemic immunity that would improve the clinical response of the primary tumour and eradicate residual micrometastatic disease [23]. Pre-clinical work suggests that neoadjuvant depletion of regulatory T cells (Treg) in mice led to a lack of observable lung micrometastases [24]. Compared to mice that received adjuvant Treg depletion, mice with neoadjuvant treatment had improved overall survival. In the clinical setting, the administration of two pre-operative doses of a PD-1 inhibitor in patients with resectable non-small cell lung cancer led to an increased number of T-cell clones in the tumour and peripheral blood [25]. Similarly, in the OpACIN trial, where patients with palpable stage III melanoma were randomised to receive adjuvant (four courses after surgery) or neoadjuvant (two courses before and two after surgery) treatment with ipilimumab and nivolumab [26], investigators reported higher rates of tumour-resident T-cell clones in the peripheral blood of patients who received neoadjuvant compared to patients who received adjuvant immunotherapy. In the updated outcome analysis, the 3-year relapse-free survival and OS rates were 80% and 90% for the neoadjuvant arm and 60% and 67% for the adjuvant arm, respectively. The study was not powered to assess differences between arms [27].

Chemotherapy agents are evaluated to identify the best partner to be combined with immunotherapy, using different administration schemes, including induction chemotherapy followed by immunotherapy or induction immunotherapy followed by a combination of chemotherapy and immunotherapy. In metastatic TNBC, the addition of atezolizumab to nab-paclitaxel [22] but not to paclitaxel [28] improved the PFS in metastatic TNBC suggesting that the chemotherapy partner might be important for the efficacy of atezolizumab. There are pre-clinical data suggesting synergistic effects between specific chemotherapeutic agents and immunotherapy including stimulation of anti-tumour immune responses by chemotherapy [29–35]. These data support the upregulation of immune-related genes, increased T-cell infiltration and T-cell receptor diversity, recruitment of functional dendritic cell-like antigen-presenting cells into the tumour bed and enhancement of the efficacy of immunotherapy.

In this article, we review the literature on neoadjuvant clinical trials evaluating immunotherapy in breast cancer. We discuss the main findings of the reported studies and the need for biomarker discovery to individualise treatment selection (Tables 1 and 2). Ongoing clinical trials are reported as well.

## Neoadjuvant immunotherapy trials in early-stage breast cancer

### Efficacy

One of the first trials evaluating neoadjuvant treatment with immunotherapeutic agents was the I-SPY 2 trial (Investigation of Serial Studies to Predict Your Therapeutic Response With Imaging and Molecular Analysis 2), a multi-centre, phase 2, randomised clinical trial [17]. Based on the adaptive design, therapeutic agents that would not prove to be efficacious would be dropped from the study, while others showing clinical benefit would quickly move forward to phase III clinical trials. In multiple concurrently enrolling therapeutic arms, the efficacy of novel drugs in combination with standard neoadjuvant chemotherapy was compared to standard treatment alone. One of the arms evaluated the benefit from the addition of the programmed cell death protein 1 (PD-1) inhibitor, pembrolizumab to weekly paclitaxel (for 12 weeks) followed by four cycles of doxorubicin and cyclophosphamide every 3 weeks, in patients with TNBC or hormone-receptor-positive, HER2-negative, early-stage breast cancer. Sixty-nine patients were randomised to receive pembrolizumab. The addition of pembrolizumab to standard neoadjuvant therapy resulted in increased pCR rates in patients with HER2-negative tumours (estimated pCR rates with pembrolizumab: 44%, 95% probability interval: 33–55 versus 17% without pembrolizumab, 95% probability interval: 11–23) [17]. In patients with TNBC, there was a threefold increase in pCR rates with the addition of pembrolizumab (22% without pembrolizumab versus 60% with pembrolizumab). The addition of pembrolizumab resulted in a similar improvement of pCR rates in hormone receptor-positive, HER2-negative disease (30%–13%). The pCR rate was less-than-expected in the control arm of the trial (22% in the TNBC subgroup), compared to previous studies reporting higher pCR rates ranging from 26% to 38% with standard chemotherapy [36, 37]. Therefore, the statistically significant increase of pCR rates with the addition of immunotherapy to standard treatment needs to be interpreted with caution. A newer investigational arm in the I-SPY 2 trial is evaluating the benefit from adding the combination of the PD-L1 inhibitor, durvalumab and the poly ADP ribose polymerase inhibitor, olaparib to standard neoadjuvant chemotherapy (NCT01042379).

Apart from I-SPY 2, pembrolizumab was evaluated in combination with chemotherapy at the neoadjuvant setting in other trials [18, 19]. KEYNOTE-173 was a multi-cohort phase 1b study assessing different time and dosing schedules of six neoadjuvant chemotherapy regimens (paclitaxel, nab-paclitaxel, doxorubicin, cyclophosphamide and carboplatin) combined with pembrolizumab in patients with locally advanced TNBC (NCT02622074) [18]. Overall, the pCR rate was 60% (90% CI: 30–85) [18]. Patients who had achieved pCR had 12-month event-free survival (EFS) rate of 100% compared to patients who had not (88%).

Based on these preliminary promising data, the addition of pembrolizumab to neoadjuvant chemotherapy was prospectively evaluated in the KEYNOTE-522 phase III clinical trial [19]. This was the first prospective phase III, randomised, double-blind trial assessing neoadjuvant chemotherapy combined with pembrolizumab versus placebo, followed by adjuvant treatment with pembrolizumab/placebo in order to complete 1 year in patients with early-stage TNBC. The trial enrolled patients with a node-positive disease or with tumours > 2 cm irrespective of nodal status. Patients were stratified based on nodal status (positive or negative), tumour size (T1/T2 or T3/T4) and administration schedule of carboplatin (weekly or every-3-week). Chemotherapy comprised of four cycles of carboplatin and paclitaxel followed by four cycles of doxorubicin/epirubicin and cyclophosphamide. The primary endpoints of the study were pCR rates as assessed locally and EFS in the intention-to-treat population. At the first interim analysis including 602 patients, there was an impressive increase in pCR rates from 51.2% in the placebo-arm to 64.8% in pembrolizumab-arm (*p* < 0.001) [19]. The benefit from the addition of pembrolizumab to chemotherapy was maintained in all clinical subgroups. At a median follow-up of 15.5 months, 7.4% of patients who received pembrolizumab had recurred compared to 11.8% of patients who received placebo (HR = 0.63, 95% CI: 0.43–0.93). Because statistical significance at the pre-specified p value boundary of 0.000051 was not reached, a longer follow-up is needed [19]. Long-term safety and efficacy data are eagerly awaited.

Two smaller neoadjuvant studies failed to demonstrate a statistically significant increase in pCR rates when the PD-L1 inhibitor durvalumab [20] and atezolizumab [21], respectively, were added to neoadjuvant chemotherapy. GeparNuevo was a prospective, randomised, double-blind, placebo-controlled phase II trial that evaluated the improvement in pCR rates by the addition of durvalumab in neoadjuvant treatment of patients with non-metastatic breast cancer (NCT02685059) [20]. Patients received neoadjuvant chemotherapy with nab-paclitaxel followed by dose-dense epirubicin/cyclophosphamide concurrently with durvalumab versus placebo. Of the 235 patients screened, 174 patients received treatment. pCR was noted at 53% of patients who received durvalumab compared to 44% of patients who received placebo (unadjusted continuity corrected *p* = 0.287). Intriguingly, there was a significant improvement in pCR rates in patients who received durvalumab two weeks before the start of chemotherapy (window-phase) compared to placebo (pCR rate 61% versus 41%, OR = 2.22, 95% CI 1.06–4.64, *p* = 0.035; interaction *p* = 0.048). However, currently, it cannot be concluded whether this difference is due to immunological priming or due to a chance subgroup analysis finding. This interesting observation needs further validation. Moreover, preliminary results from the phase III NeoTRIPaPDL1 Michelangelo study showed that the addition of atezolizumab to neoadjuvant treatment with carboplatin and nab-paclitaxel in patients with non-metastatic TNBC resulted in not significantly different pCR rates compared with chemotherapy alone (43.5% versus 40.8%, respectively) [21]. However, the study’s primary endpoint was EFS at 5 years following randomization of the last patient. Differences in clinical outcomes between the GeparNuevo, the NeoTRIPaPDL1 Michelangelo and the Keynote-522 study [19, 21] might be related to different drugs (PD-L1 versus PD-1 inhibitors, respectively), different chemotherapy backbone regimens and differences in the trials’ sample size. Ongoing phase III trials (NCT03281954, NCT03498716) will clarify the role of atezolizumab in early TNBC. Details on the efficacy and toxicity of the aforementioned schemes are depicted in Tables 1 and 2, respectively.

More recently, the IMpassion031, a phase III, randomised, double-blind study, evaluating the use of immunotherapy in early-stage TNBC, met its primary endpoint [38]. Overall, 333 patients were randomised to receive neoadjuvant chemotherapy (nab-paclitaxel followed by doxorubicin and cyclophosphamide) combined with atezolizumab versus chemotherapy compared to placebo and chemotherapy, followed by maintenance therapy with atezolizumab. The primary endpoint was pCR in the intention-to-treat population and in the PD-L1-positive population. There was a significant increase in the pCR rate in patients who received atezolizumab compared to patients who received placebo, regardless of PD-L1 expression [38].

Except for I-SPY 2 trial that enrolled patients with high-proliferative, hormone-receptor-positive tumours in addition to TNBC, all the rest of the aforementioned trials focused on triple-negative disease. However, the intriguing findings of clinical benefit in high-proliferative hormone receptor-positive disease along with preliminary data of immunotherapeutic benefit in metastatic HER2-positive disease [39] highlight the interest to further evaluate the role of immunotherapy in other breast cancer subtypes.

### Adverse events

Studies on neoadjuvant immunotherapy combined with chemotherapy showed that these combinations are relatively well-tolerated and do not compromise the administration of treatment. Discontinuation rates were similar between patients who received immunotherapy versus placebo [19–21] (Table 2). Serious treatment-related adverse events were observed in 40% (24 of 60) of patients in KEYNOTE-173 [18] and 34% (59 of 174) of patients in GeparNuevo (30 patients who received durvalumab and 29 who received placebo) [20]. Importantly, in KEYNOTE-522, a higher rate of serious adverse events was reported in 33% of patients who received pembrolizumab combined with chemotherapy compared to 20% of patients who received chemotherapy alone [19]. The majority of adverse events were attributed to chemotherapy. All grade immune-related toxicity was reported in 30% to 42% of patients [18, 19]. Most common immune-related adverse events were associated with thyroid dysfunction [17, 19, 20]. Other common immune-related adverse events included colitis, pneumonitis, skin reactions and adrenal insufficiency. Grade 5 immune-related adverse events were not reported in most neoadjuvant trials [17, 18, 20]. However, there were three deaths in the pembrolizumab group, attributed to pneumonitis, pulmonary embolism and sepsis, reported in KEYNOTE-522 trial [19].

The safety profile reported in neoadjuvant studies with immunotherapy is in line with data from trials in the metastatic setting. Currently available data show that immunotherapy can be combined with chemotherapy in the neoadjuvant setting without compromising the administration of standard treatment and completion of surgery. However, these findings should be interpreted with caution. The administration of immunotherapeutic agents may be associated with irreversible toxicities, and therefore, longer follow-up data to establish the long-term safety of immunotherapy in the curative setting are warranted.

## Immunotherapy biomarkers

Immunotherapy has changed the therapeutic landscape of cancer, providing improved clinical outcomes and long-term survival compared to chemotherapy agents in selected patients. However, not all patients benefit from this treatment, while a proportion of patients experiences serious adverse events. It is critical to identify predictive biomarkers of response, to accurately select patients who will benefit from immunotherapy and spare the rest from unnecessary toxicity and costs [40]. Various molecular alterations and immunological parameters have been suggested to predict benefit or resistance from immunotherapeutic agents in diverse tumour subtypes [41–45]. Neoadjuvant clinical trials provide an ideal setting for biomarker identification and validation. Thus, in patients with breast cancer receiving neoadjuvant immunotherapy, several biomarkers are being evaluated for their association with response to PD-1 or PD-L1 inhibitors, including PD-L1 expression and tumour-infiltrating lymphocytes (TILs) [20, 46].

Positive PD-L1 expression is currently used for selecting patients with metastatic TNBC for first-line treatment with atezolizumab and nab-paclitaxel [22]. Similarly, in the Keynote-355 study, the benefit of adding pembrolizumab was observed in PD-L1-positive patients only [47]. PD-L1 expression was assessed with different antibodies using different cut-offs. In IMpassion-130 trial, PD-L1 expression was assessed using the SP142 PD-L1 immunohistochemical assay (Ventana Medical Systems), while PD-L1 positivity was defined as the presence of stained tumour-infiltrating immune cells covering ≥ 1% of the tumour area [22]. On the contrary, PD-L1 protein expression in KEYNOTE-355 was determined using Combined Positive Score (CPS) (PD-L1 IHC 22C3 pharmDx, Agilent, Santa Clara, CA), defined as the number of PD-L1 staining cells (tumour cells, lymphocytes and macrophages) divided by the total number of viable tumour cells, multiplied by 100 [47]. Tumour was considered positive if CPS ≥ 1. Despite the benefit seen in patients with PD-L1-positive advanced TNBC with the addition of immunotherapy to chemotherapy, there are no data that PD-L1 status can be used for selection of patients with early TNBC who will benefit from the addition of checkpoint inhibitors to neoadjuvant chemotherapy. In early-stage breast cancer trials evaluating the use of immunotherapy in the neoadjuvant setting, PD-L1 positivity was defined using different antibodies. In KEYNOTE-522 and KEYNOTE-173 trials, PD-L1 expression was assessed using the IHC 22C3 pharmDx assay CPS [18, 19]. Tumours with PD-L1 expression ≥1% were defined as positive. PCR rates were high with the addition of pembrolizumab, irrespectively of PD-L1 expression levels [19]. In KEYNOTE-522, patients with PD-L1-positive tumours, pCR rates were 68.9% for those in the pembrolizumab arm and 54.9% for those in the placebo arm. In patients with PD-L1–negative tumours, pCR rates were 45.3% for those who received pembrolizumab and chemotherapy and 30.3% for those who received placebo and chemotherapy. Among the 60 patients enrolled in KEYNOTE-173, pre-treatment PD-L1 CPS was assessed for 52 (87%) [18]. PD-L1 CPS was associated with higher pCR rates (area under the receiver operating characteristic curve (AUROC) = 0.658). Specifically, pCR rates were 60% versus 40% in patients with PD-L1 CPS ≥ 1 and <1, respectively. In the GeparNuevo trial, PD-L1 expression was evaluated using the Ventana SP263 antibody (Ventana Medical Systems Inc., Tucson, AZ) and was defined as the proportion of tumour cells with membranous staining (PD-L1-TC) and proportion of TILs with membranous or cytoplasmic staining (PD-L1-IC) [20]. If any of these proportions were ≥1%, tumours were considered PD-L1 positive. Details on the assays used for PD-L1 expression is shown in Table 3. Even though pCR rates were higher in patients with PD-L1 positive compared to PD-L1-negative tumours (54.3% versus 30.0%, respectively, *p* = 0.048), PD-L1 expression did not predict for response to durvalumab [20]. Patients who received durvalumab had numerically higher pCR rates when tumours were PD-L1-positive compared to PD-L1-negative (58.0% versus 44.4%, respectively, *p* = 0.445); however, this difference was not statistically significant. Despite differences in the assessment of PD-L1 expression regarding the use of diverse antibodies and scoring systems, this biomarker does not seem to be predictive for response to immunotherapy in the neoadjuvant setting.

Increased TIL concentration has been identified as a predictor of response to neoadjuvant chemotherapy in all molecular subtypes of breast cancer [48, 49]. In addition, there are data suggesting a prognostic effect for TIL levels varying according to the tumour subtype [48, 50–53]. A pooled analysis of six randomised trials including 3771 patients with breast cancer who received neoadjuvant combination chemotherapy demonstrated that patients with high TILs had higher rates of pCR, irrespective of the tumour subtype [48]. Importantly, the increase in TILs was associated with longer overall survival in patients with TNBC, but not in patients with HER2-positive (no difference) or hormone receptor-positive/HER2-negative tumours (shorter overall survival). Whether this immunologic parameter can be used as a biomarker predicting response to immunotherapy is under investigation [19]. Preliminary data from GeparNuevo trial showed that stromal TILs were not predictive for durvalumab response [20]. In fact, baseline stromal TILs (sTILs) predicted higher pCR rates not only in patients who received durvalumab but also in patients who received placebo. Interestingly, during the window-phase of the trial, the increase of intratumoural TILs, from baseline to post-window samples, was independently associated with high pCR rates in the durvalumab group (OR 9.36, 95% CI 1.26–69.65, *p* = 0.029), but not in patients who received placebo (OR 1.22, 95% CI 0.65–2.27, *p* = 0.540). In KEYNOTE-173, of 60 patients who enrolled in the trial, sTILs were assessed for 53 (88%) patients before treatment and for 49 patients (82%) after one dose of pembrolizumab [18]. Median pre-treatment and on-treatment sTIL levels were higher for patients with pCR. In addition, higher pre-treatment and on-treatment sTILs were associated with higher pCR rates (AUROC 0.653 and 0.690, respectively). The correlation between PD-L1 CPS and sTILs (pre- and on-treatment) was moderate to strong. These preliminary data suggest that, while there seems to be an association of high levels of TILs with high pCR rates, TILs cannot be used alone as a biomarker predicting response to immunotherapy. Further research is warranted to determine the optimal cut-offs or the use of TIL expression as a continuous variable to form a standardised methodology for evaluating TILs.

Moreover, high expression of immune-related gene signatures has been associated with higher pCR rates to neoadjuvant anthracycline-/taxane-based chemotherapy [54]. High levels of immune-related gene signatures have been associated with high levels of TILs [55]; however, the role of immune-related gene signatures to predict benefit from immunotherapy is unclear.

## New strategies for immunotherapy trials in early-stage breast cancer

There is a significant number of clinical trials evaluating the clinical benefit from immunotherapeutic agents administered as neoadjuvant treatment of patients with breast cancer (Table 4). In these trials, various immunotherapeutic agents are administered often in combination with chemotherapy. 

Other clinical trials evaluate combinations of immunotherapy with targeted agents (i.e., CDK4/6 inhibitors, anti-HER2 agents). For instance, CDK4/6 inhibitors have been shown to enhance anti-tumour immune response, thus acting synergistically with immunotherapeutic agents [56–58]. In addition, several trials are evaluating the benefit and safety of vaccines, either against personalised cancer cell epitopes or commonly overexpressed proteins, such as HER2. Other trials are assessing PD-1/PD-L1 inhibitors in combination with other immunotherapy agents, intratumoural injections of immunotherapeutic agents (oncolytic viruses) or *ex vivo* expanded activated T cells. Other interesting approaches are the use of cryoablation [59] or stereotactic radiotherapy [60] to render a tumour immunogenic, as well as an in situ tumour vaccine or the use of anti-CD73 antibodies to block the adenosine pathway.

A significant proportion of immunotherapy trials has initially focused on TNBC, possibly due to high PD-L1 expression and TIL levels in those tumours compared to other subtypes. Nevertheless, a number of immunotherapy trials have been initiated in high-proliferative hormone receptor-positive/HER2-negative and HER2-positive tumours (Table 4).

## Perspectives and conclusions

Neoadjuvant trials represent valuable platforms to test the efficacy of innovative drugs and/or a combination of treatments and evaluate the predictive value of new biomarkers. The PD-1 inhibitor pembrolizumab combined with neoadjuvant chemotherapy significantly increased the pCR rates in patients with TNBC. Long-term outcome data from this trial are eagerly awaited. Ongoing trials are testing other PD-1/PD-L1 inhibitors in combination with neoadjuvant chemotherapy in TNBC. Preliminary data show that the addition of immunotherapy to neoadjuvant chemotherapy might also be active in other tumour subtypes beyond TNBC. However, not all clinical trials demonstrated significant differences in clinical outcomes with the addition of immunotherapy to neoadjuvant chemotherapy. These differences might be related to patient selection, trials’ sample size, use of different immunotherapy drugs (PD-L1 versus PD-1 inhibitors), different chemotherapy backbone regimens and/or continuation of immunotherapy after curative surgery.

In addition to chemotherapy, which has been shown to increase tumour immunogenicity, other targeted agents such as anti-HER2 agents or the CDK46 inhibitors or other immunotherapeutic agents are being tested in combination with PD-1/PD-L1 inhibitors. Other agents, including bevacizumab, have been shown to increase the pCR rates without any difference in long-term outcomes. Therefore, we still need to determine whether pCR after neoadjuvant immunotherapy combined with chemotherapy is associated with improved long-term outcomes. Existing biomarkers, including PD-L1, seem to be ineffective in the neoadjuvant setting for the accurate selection of patients who will benefit from checkpoint blockade. Immunotherapy is associated with potentially irreversible toxicities and prohibitive costs. Therefore, it is critical to identify patients, where the escalation of treatment is required to improve outcomes, along with robust predictive biomarkers of efficacy and toxicity, to select patients who will benefit from the addition of neoadjuvant immunotherapy, thus sparing the rest from an ineffective treatment with unnecessary toxicity and treatment costs. Ultimately, patients with early-stage breast cancer will receive individualised management based on their tumour clinicopathological, molecular and immune-related characteristics.

## Conflicts of interest

E.F.: Stock ownership: GENPREX INC, ARIAD, Deciphera Pharmaceuticals, Inc. Travel grant: Merck, Pfizer and K.A.M Oncology/Hematology. Advisory: LEO Pharma. Speaker fees: Roche, Pfizer

M.I.: Consultant or advisory role: Celgene, Novartis, Pfizer, Seattle Genetics, Tesaro; Speaker Honoraria: Novartis; travel grants: Pfizer, Amgen; research grants to my Institute: Roche, Menarini Silicon Biosystems, Janssen Diagnostics, Pfizer.

## Financial conflicts of interest in relation to this manuscript

The authors have no financial conflicts of interest.

## Funding statement

The authors did not receive any funding for the preparation of this review article.

## Figures and Tables

**Table 1.** Reported neoadjuvant clinical trials with PD-1 and PD-L1 inhibitors in early-stage breast cancer: design and Outcomes.

Year First/Last author	Trial name	Trial type	Tumour subtype	Treatment arms	Immunotherapeutic agent	Primary endpoints	No of pts treated (N)	Reported biomarker(s)	Primary outcome	Secondary outcome
202017[17] Nanda Esserman	I-SPY 2	Randomised, adaptive design, phase II, platform trial	TNBC (114 pts) and HR-positive/ HER2-negative (136 pts)	Paclitaxel followed by doxorubicin and cyclophosphamide with or without pembrolizumab for four cycles concurrently with paclitaxel	Pembrolizumab (anti-PD-1)	pCR	25049 (69 with pembrolizumab and 181 without pembrolizumab)	No info	pCR rates: Pembrolizumab versus no-pembrolizumab: HER2-negative: 44% versus 17%, HR-positive/HER2-negative: 30% versus 13%, TNBC: 60% versus 22%	Residual cancer burden, 3-year event-free survival 3-year distant recurrence-free survival
2019[20] Loibl, Schneeweiss	GeparNuevo	Phase II, randomised, double-blind, placebo-controlled	TNBC	Durvalumab or placebo with nab-paclitaxel followed by epirubicin and cyclophosphamide	Durvalumab (anti-PD-L1)	pCR	174	TILs PD-L1	pCR rates: 53.4% with durvalumab versus 44.2% with placebo	Secondary pCR end points Clinical response in the breast and nodes after taxane treatment and before surgery Toxicity
202019[19] Schmid O’Shaughnessy	KEYNOTE-522	Phase III, randomised, double-blind, placebo-controlled	TNBC	Paclitaxel and carboplatin plus pembrolizumab or placebo followed by doxorubicin or epirubicin and cyclophosphamide with pembrolizumab or placebo. Maintenance after surgery with pembrolizumab or placebo for up to nine cycles	Pembrolizumab (anti-PD-1)	pCR and event-free survival in the ITT population	1174 (784 patients in pembrolizumab–chemotherapy group and 390 patients in the placebo–chemotherapy group)	TILs PD-L1	pCR rates: 64.8% in pembrolizumab-arm versus 51.2% in the placebo-arm (*p* = 0.00055)	18-month OS: 91.3% (95% CI, 88.8 to 93.3) in the pembrolizumab– Arm and 85.3% (95% CI, 80.3 to 89.1) in the placebo arm the median was not reached in either group
2020[18] Schmid Loi	KEYNOTE-173	Phase 1b, 6-cohort	TNBC	6 neoadjuvant chemotherapy regimens (paclitaxel, nab-paclitaxel, doxorubicin, cyclophosphamide, carboplatin) combined with pembrolizumab	Pembrolizumab (anti-PD-1)	Safety and a recommended phase II regimen	60 (10 per cohort)	PD-L1 CPS, sTILs	Safety (see Table 2)	pCR rate in all subgroups: 60% (90% CI 49% to 71%) 12-month EFS rate: 100% for patients who achieved pCR versus 88% in patients who did not
2019[21] Gianni Viale	NeoTRIPaPDL1 Michelangelo	Randomised, parallel assignment, open label	TNBC	Carboplatin and abraxane with or without atezolizumab for 8 cycles followed by surgery and by 4 anthracycline-based cycles	Atezolizumab (anti-PD-L1)	Event-free survival 5 years after randomization of the last patient	280	PD-L1	EFS-not reported yet	pCR rates: 43.5% with atezolizumab versus 40.8% without atezolizumab (*p* = 0.066)
2020[38] Mittendorf Harbeck	IMpassion031	Phase III, randomised, double-blind, placebo-controlled	TNBC	Nab-paclitaxel for 12 weeks followed by doxorubicin and cyclophosphamide for 8 weeks with or without atezolizumab	Atezolizumab (anti-PD-L1)	pCR in the ITT population and PD-L1-positive populations.	333 (165 patients in atezolizumab–chemotherapy group and 168 patients in the placebo–chemotherapy group)	PD-L1	pCR rates ITT population: 58% (95 of 165) with atezolizumab versus 41% (69 of 168) with placebo, one-sided *p* = 0·0044 PD-L1-positive population: 69% with atezolizumab versus 49% with placebo, one-sided *p* = 0·021	Results were immature for event-free survival, disease-free survival or overall survival

CPS: combined positive score, HER2: human epidermal growth factor receptor 2, HR: hormone receptor, ITT: intention-to-treat, pCR: pathologic complete response, N: number, PD-1: programmed cell death 1, PD-L1: programmed cell death ligand 1, sTILs: stromal tumour-infiltrating lymphocytes, TIL: tumour-infiltrating lymphocyte, TNBC: triple-negative breast cancer

**Table 2.** Toxicity of neoadjuvant immunotherapy regimens in early-stage breast cancer trials.

	AE	Serious treatment-related AE	Discontinuation rates due to AE	Immune-related AE
I-SPY 2	Common AE in pembrolizumab arm: Fatigue: 87% Nausea 79.9% Diarrhoea: 56.5% Sensory neuropathy: 56.5%			The most common immune-related AE was thyroid dysfunction (hypothyroidism and hyperthyroidism) in 13% (9 of 69 patients) who received pembrolizumab
GeparNuevo	AEs were similar between two groups (durvalumab versus placebo), with the exception of thyroid dysfunction (more frequent in durvalumab-arm)	30 (32.6%) in the durvalumab and 29 (35.4) in the placebo arm	Similar discontinuation rates between patients who received durvalumab versus placebo Durvalumab was discontinued in 20 (of 92) patients in durvalumab arm compared with 17 (of 82) patients on placebo arm	Thyroid dysfunction Hyperthyroidism more common in durvalumab-arm (9.8 versus 1.2%)
KEYNOTE-522	99.0% (of 781 patients) in the pembrolizumab arm and 99.7% (of 389) in the placebo arm	32.5% (pembrolizumab arm) and 19.5% (placebo arm)	23.3% (pembrolizumab arm) and 12.3% (placebo arm)	Hypothyroidism 13.7% (pembrolizumab) versus 3.3% (placebo) Hyperthyroidism 4.6% (pembrolizumab) versus 1% (placebo) AE of interest, grade ≥ 3, in ≥10 patients was adrenal insufficiency (in 1.3%) in the pembrolizumab–group
KEYNOTE-173	100% (60 patients)	90% (54 of 60 patients)	27% (16 of 60 patients)	30% (18 of 60 patients)
NeoTRIPaPDL1 Michelangelo	97.8% (of 138 patients) in the atezolizumab arm and 98.6% (of 140) in the chemotherapy arm	18.1% (atezolizumab arm) and 5.7% (chemotherapy arm)	25.4% (atezolizumab arm) and 25% (chemotherapy arm)	Atezolizumab arm: Hypothyroidism: 5.8% Hyperthyroidism:0.7% Colitis:1.5% Pacreatitis:1.5%
Impassion031	99% (atezolizumab arm) and 99% (chemotherapy arm)	23% (atezolizumab arm) and 16% (chemotherapy arm)	23% (atezolizumab arm) and 20% (chemotherapy arm)	18.1% (atezolizumab arm) and 5.7% (chemotherapy arm)

AE: adverse events, EFS: event-free survival, HER2: human epidermal growth factor receptor 2, HR: hormone receptor, pCR: pathologic complete response, TNBC: triple-negative breast cancer

**Table 3.** Comparison of PD-L1 used in early-stage breast cancer.

Antibody	Developer	Cut-off	Drug	Clinical trial
IHC 22C3 pharmDx assay	Agilent, Santa Clara, California	CPS≥1%	Pembrolizumab	KEYNOTE-522 KEYNOTE-173
IHC SP263 assay	Ventana Medical Systems Inc., Tucson, Arizona	Proportion of tumour cells with membranous staining and/or proportion of TILs with membranous or cytoplasmic staining ≥ 1%,	Durvalumab	GeparNuevo
IHC SP142 assay	Ventana Medical Systems Inc., Tucson, Arizona	Immune cells≥1%	Atezolizumab	Impassion031

CPS: combined positive score, IHC: immunohistochemistry, TILs: tumour-infiltrating lymphocytes

**Table 4.** Ongoing neoadjuvant trials with immunotherapeutic agents.

Trial name	Immunotherapeutic agent	Tumour subtype	Trial type	Identifier
**Trials with PD-1/PD-L1 inhibitors and chemotherapy**				
PHOENIX DDR/Anti-PD-L1 Trial	Durvalumab	TNBC	Window of opportunity	NCT03740893
Safety and Efficacy of Durvalumab Combined to Neoadjuvant Chemotherapy in Localised Luminal B HER2(-) and Triple-Negative Breast Cancer (B-IMMUNE)	Durvalumab	HR-positive, HER2-negative	Phase Ib/II, open label	NCT03356860
Neoadjuvant MEDI4736 Concomitant With Weekly Nab-paclitaxel and Dose-dense AC for Stage I-III Triple-Negative Breast Cancer	Durvalumab	TNBC	Phase I/II, single arm	NCT02489448
Study of Immunotherapy in Combination With Chemotherapy in HER2-negative Inflammatory Breast Cancer (PELICAN)	Pembrolizumab	HER2-negative	Phase II randomised open-label	NCT03515798
Neoadjuvant Pembrolizumab + Decitabine Followed by Std Neoadj Chemo for Locally Advanced HER2- Breast Ca	Pembrolizumab	HER2-negative	Phase II, 2-cohort, open-label	NCT02957968
Neoadjuvant Phase II Study of Pembrolizumab And Carboplatin Plus Docetaxel in Triple-Negative Breast Cancer (NeoPACT)	Pembrolizumab	TNBC	Phase II, open label	NCT03639948
Carboplatin and Paclitaxel With or Without Atezolizumab Before Surgery in Treating Patients With Newly Diagnosed, Stage II-III Triple-Negative Breast Cancer	Atezolizumab	TNBC	Phase II, randomised, open label	NCT02883062
Nab-Paclitaxel and Atezolizumab Before Surgery in Treating Patients With Triple-Negative Breast Cancer	Atezolizumab	TNBC	Phase II, open label	NCT02530489
Pembrolizumab in Treating Patients With Hormone Receptor-Positive, Localised Inflammatory Breast Cancer Who Are Receiving Hormone Therapy and Did Not Achieve a Pathological Complete Response to Chemotherapy	Pembrolizumab	HR-positive, HER2-negative	Phase II, open label	NCT02971748
Pembrolizumab in Treating Patients With Triple-Negative Breast Cancer	Pembrolizumab	TNBC	Phase III, randomised, open label	NCT02954874
Neoadjuvant Pembrolizumab(Pbr)/Nab-Paclitaxel Followed by Pbr/Epirubicin/Cyclophosphamide in TNBC (NIB)	Pembrolizumab	TNBC	Phase II, one-arm, open-label	NCT03289819
Study of Pembrolizumab (MK-3475) Versus Placebo in Combination With Neoadjuvant Chemotherapy & Adjuvant Endocrine Therapy in the Treatment of Early-Stage Oestrogen Receptor-Positive, Human Epidermal Growth Factor Receptor 2-Negative (ER+/HER2-) Breast Cancer (MK-3475-756/KEYNOTE-756)	Pembrolizumab	HR-positive, HER2-negative	Phase III, randomised, double-blind	NCT03725059
Trial of Nivolumab With Chemotherapy as Neoadjuvant Treatment in Inflammatory Breast Cancer	Nivolumab	TNBC or HR-positive, HER2-negative	Phase II, open label	NCT03742986
Study of Nivolumab Versus Placebo in Participants With High-Risk Breast Cancer (CheckMate 7FL)	Nivolumab	HR-positive, HER2-negative	Phase III, randomised, double-blind, placebo-controlled	NCT04109066
Clinical Trial of Neoadjuvant Chemotherapy With Atezolizumab or Placebo in Patients With Triple-Negative Breast Cancer Followed After Surgery by Atezolizumab or Placebo	Atezolizumab	TNBC	Phase III, randomised, double-blind,	NCT03281954
Neoadjuvant Treatment of HER2-Positive Early High-Risk and Locally Advanced Breast Cancer (APTneo)	Atezolizumab	HER2-positive	Phase III, randomised, open label	NCT03595592
A Study to Investigate Atezolizumab and Chemotherapy Compared With Placebo and Chemotherapy in the Neoadjuvant Setting in Participants With Early-Stage Triple-Negative Breast Cancer (IMpassion031)	Atezolizumab	TNBC	Phase III, double-blind, randomised, placebo-controlled	NCT03197935
**Trials with PD-1/PD-L1 inhibitors and CDK4/6 inhibitors**				
Neoadjuvant Study of Abemaciclib, Durvalumab and an Aromatase Inhibitor Early-Stage Breast Cancer	Durvalumab	HR-positive, HER2-negative	Early Phase I	NCT04088032
A Study of Neoadjuvant Nivolumab + Abemaciclib or Palbociclib + Anastrozole in Post-Menopausal Women and Men With Primary Breast Cancer (CheckMate 7A8)	Nivolumab	HR-positive, HER2-negative	Phase II, randomised, non-comparative, multi-arm,	NCT04075604
Neoadjuvant Tamoxifen, Palbociclib, Avelumab in Oestrogen Receptor-Positive Breast Cancer (ImmunoADAPT)	Avelumab	HER2-positive	Phase II, open label	NCT03573648
**Trials with PD-1/PD-L1 inhibitors and anti-HER2 treatment**				
TAHP for Patients With HER2-positive Early Breast Cancer and Subsequent AHP Adjuvant Therapy After Surgery	Atezolizumab	HER2-positive	Phase IB-II	NCT03881878
Atezolizumab in combination with trastuzumab emtansine or with trastuzumab and pertuzumab in patients with HER2-positive breast cancer and atezolizumab with doxorubicin and cyclophosphamide in HER2-negative breast cancer	Atezolizumab	HER2-positive	Phase Ib, multi-cohort	NCT02605915
Neoadjuvant Her2-targeted Therapy and Immunotherapy With Pembrolizumab	Pembrolizumab	HER2-positive	Phase II open-label, randomised	NCT03747120
A Study With Pembrolizumab in Combination With Dual Anti-HER2 Blockade With Trastuzumab and Pertuzumab in Early Breast Cancer Patients With Molecular HER2-enriched Intrinsic Subtype (Keyriched-1)	Pembrolizumab	HER2-positive	Phase II, prospective, single arm, open label	NCT03988036
A Study To Evaluate the Efficacy and Safety Of Atezolizumab or Placebo in Combination With Neoadjuvant Doxorubicin + Cyclophosphamide Followed By Paclitaxel + Trastuzumab + Pertuzumab In Early Her2-Positive Breast Cancer (IMpassion050)	Atezolizumab	HER2-positive	Phase III, randomised, double-Blind, placebo-controlled	NCT03726879
Improving Pre-operative Systemic Therapy for Human Epidermal Growth Factor Receptor 2 (HER2) Amplified Breast Cancer (PREDIX II HER2)	Atezolizumab	HER2-positive	Phase II, randomised, open label	NCT03894007
**Trials with PD-1/PD-L1 inhibitors and Radiation treatment**				
Neo-adjuvant Chemotherapy Combined With Stereotactic Body Radiotherapy to the Primary Tumour +/- Durvalumab +/- Oleclumab in Luminal B Breast Cancer: (Neo-CheckRay)	Durvalumab, Oleclumab	Luminal B	Phase II, randomised, open-label	NCT03875573
Breast Cancer Study of Pre-Operative Pembrolizumab + Radiation	Pembrolizumab	TNBC or HR-positive, HER2-negative	Phase I/II, single arm with two cohorts	NCT03366844
**Trials with vaccines**				
HER2 Directed Dendritic Cell Vaccine During Neoadjuvant Therapy of HER2+Breast Cancer	Dendritic Cell Vaccine (DC1)	HER2-positive	Early Phase I	NCT03387553
HER-2 Pulsed DC Vaccine to Prevent Recurrence of Invasive Breast Cancer Post Neoadjuvant Chemotherapy	HER-2 pulsed Dendritic Cell Vaccine	HER2-positive	Phase I	NCT02061423
Safety and Immunogenicity of a Personalised Synthetic Long Peptide Breast Cancer Vaccine Strategy in Patients With Persistent Triple-Negative Breast Cancer Following Neoadjuvant Chemotherapy	Personalised synthetic long peptide vaccine	TNBC	Phase I, single-arm	NCT02427581
Safety and Immunogenicity of a Personalised Polyepitope DNA Vaccine Strategy in Breast Cancer Patients With Persistent Triple-Negative Disease Following Neoadjuvant Chemotherapy	Personalised polyepitope DNA vaccine	TNBC	Phase I open-label	NCT02348320
Safety and Immune Response to a Mammaglobin-A DNA Vaccine In Breast Cancer Patients Undergoing Neoadjuvant Endocrine Therapy	Mammaglobin-A DNA Vaccine	HR-positive, HER2-negative	Phase IB	NCT02204098
Vaccination of High-Risk Breast Cancer Patients	Chemovax	HR-positive	Phase I/II study, single-arm	NCT02229084
Phase II Trial of Combination Immunotherapy With NeuVax and Trastuzumab in High-risk HER2+ Breast Cancer Patients	NeuVax vaccine	HER2-positive	Phase II, prospective, randomised, single-blinded, placebo-controlled	NCT02297698
Vaccination of Triple-Negative Breast Cancer Patients	P10s-PADRE with MONTANIDE™ ISA 51 VG	TNBC	Phase II, randomised two-arm, open-label	NCT02938442
Folate Receptor Alpha Peptide Vaccine With GM-CSF in Patients With Triple-Negative Breast Cancer	Low dose FRα vaccine	TNBC	Phase II , randomised	NCT02593227
TPIV100 and Sargramostim for the Treatment of HER2-Positive, Stage II-III Breast Cancer in Patients With Residual Disease After Chemotherapy and Surgery	Vaccine Therapy	HER2-positive	Phase II, randomised	NCT04197687
**Trials with PD-1/PD-L1 inhibitors in combination with other immunotherapy drug**s				
Combination of Talimogene Laherparepvec With Atezolizumab in Early Breast Cancer (PROMETEO)	Atezolizumab, T-VEC	TNBC or HR-positive, HER2-negative	Window opportunity, single arm, exploratory	NCT03802604
M7824 in Treating Patients With Stage II-III HER2-Positive Breast Cancer	anti-PD-L1/TGFbetaRII fusion protein M7824	HER2-positive	Phase I	NCT03620201
Converting HR+ Breast Cancer Into an Individualised Vaccine (CBCV)	Pembrolizumab, CDX-301	HR-positive, HER2-negative	phase II, randomised open-label	NCT03804944
Peri-Operative Ipilimumab+Nivolumab and Cryoablation Versus Standard Care in Women With Triple-Negative Breast Cancer	Ipilimumab, nivolumab	TNBC	Phase II, randomised, open label	NCT03546686
TAC Chemotherapy and Pembrolizumab Plus Interleukin-12 Gene Therapy and L-NMMA in Triple-Negative Breast Cancer	Pembrolizumab	TNBC	Phase II, prospective, single arm, open label	NCT04095689
Durvalumab and Endocrine Therapy in ER+/Her2- Breast Cancer After CD8+ Infiltration Effective Immune-Attractant Exposure (ULTIMATE)	Durvalumab, Immune-attractant	HR-positive, HER2-negative	Phase II, open-label, single group assignment	NCT02997995
Durvalumab and Tremelimumab Before Surgery in Treating Patients With Hormone Receptor-Positive, HER2-Negative Stage II-III Breast Cancer	Durvalumab and Tremelimumab	HR-positive, HER2-negative	Early phase I	NCT03132467
Pre-operative Immunotherapy Combination Strategies in Breast Cancer (ECLIPSE)	Atezolizumab	HR-positive, HER2-negative	Phase II, open label, window of opportunity	NCT03395899
**Other immunotherapeutic approaches**				
Immunogenicity and Safety of DCs in Breast Cancer (TEBICA)	Dendritic cells	All	Phase I/II, randomised	NCT03450044
Targeted T Cells After Neoadjuvant Chemotherapy in Treating Women With Stage II or III Breast Cancer Undergoing Surgery	HER2Bi-armed activated T cells	TNBC	Phase II, open label	NCT01147016
Intratumoural TriMix Injections in Early Breast Cancer Patients (TMBA)	Trimix	All	Phase I	NCT03788083
